# Return to sport outcomes after inverted V‐shaped (IV) high tibial osteotomy were comparable to those after medial opening‐wedge high tibial osteotomy, even though the IV cohort had more severe preoperative disease

**DOI:** 10.1002/jeo2.70667

**Published:** 2026-02-23

**Authors:** Taku Ebata, Eiji Kondo, Koji Yabuuchi, Koji Iwasaki, Dai Sato, Masatake Matsuoka, Tomohiro Onodera, Kazunori Yasuda, Tomonori Yagi, Norimasa Iwasaki

**Affiliations:** ^1^ Department of Orthopaedic Surgery, Faculty of Medicine and Graduate School of Medicine Hokkaido University Hokkaido Sapporo Japan; ^2^ Centre for Sports Medicine Hokkaido University Hospital Hokkaido Sapporo Japan; ^3^ Department of Orthopaedic Surgery Yagi Orthopaedic Hospital Hokkaido Sapporo Japan; ^4^ Department of Functional Reconstruction for the Knee Joint, Faculty of Medicine Hokkaido University Hokkaido Sapporo Japan

**Keywords:** inverted V‐shaped high tibial osteotomy, knee osteoarthritis, opening‐wedge high tibial osteotomy, return to sports, Tegner activity scale

## Abstract

**Purpose:**

The primary aim was to compare return to sport (RTS) outcomes following inverted V‐shaped high tibial osteotomy (IV‐HTO) versus medial opening‐wedge (OW)‐HTO for medial knee osteoarthritis (OA). The secondary aim was to compare patient‐reported outcomes and radiographic parameters between the procedures.

**Methods:**

This retrospective cohort study included patients who underwent HTO for medial knee OA between 2017 and 2022. Inclusion criteria were preoperative sports participation and 2‐year follow‐up. Patients were allocated to IV‐HTO (IV group) or OW‐HTO (OW group) according to indications based on the planned correction angle and patellofemoral OA grade. Outcomes were assessed preoperatively and at 2 years. Between‐group comparisons used appropriate parametric/nonparametric tests, with *p* < 0.05 considered significant.

**Results:**

A total of 107 patients were analysed (IV group: 54 patients; OW group: 53 patients). Mean age was 58.4 years, body mass index (BMI) 25.6 kg/m^2^ and follow‐up 29.5 months. Preoperative femorotibial and patellofemoral Kellgren–Lawrence grades and varus deformity (% mechanical axis) were more severe in the IV group (both *p* < 0.001). RTS rates were 91% (49/54) in the IV group and 91% (48/53) in the OW group (*p* = 0.975), with mean time to RTS of 8.4 and 8.8 months, respectively (*p* = 0.523). Lysholm score and Knee Injury and Osteoarthritis Outcome Score (KOOS) improved significantly in both groups (all *p* < 0.001), with similar minimal clinically important difference (MCID) responder rates. From preoperative to postoperative assessments, OW‐HTO increased posterior tibial slope (9.3 ± 2.3° to 10.2 ± 2.4°, *p* < 0.001), whole leg length (800 ± 59 to 808 ± 59 mm, *p* < 0.001) and decreased the Caton–Deschamps ratio (1.03 ± 0.13 to 0.88 ± 0.14, *p* < 0.001), whereas IV‐HTO showed no significant changes (all *p* > 0.05).

**Conclusions:**

IV‐HTO achieved RTS and patient‐reported outcomes comparable to OW‐HTO at 2 years in patients with more severe preoperative disease.

**Level of Evidence:**

Level III, retrospective comparative study.

## Graphical abstract

Inverted V‐shaped high tibial osteotomy (IV‐HTO) achieved return to sport (RTS) and patient‐reported outcomes comparable to opening‐wedge (OW)‐HTO at 2 years in patients with more severe preoperative disease.

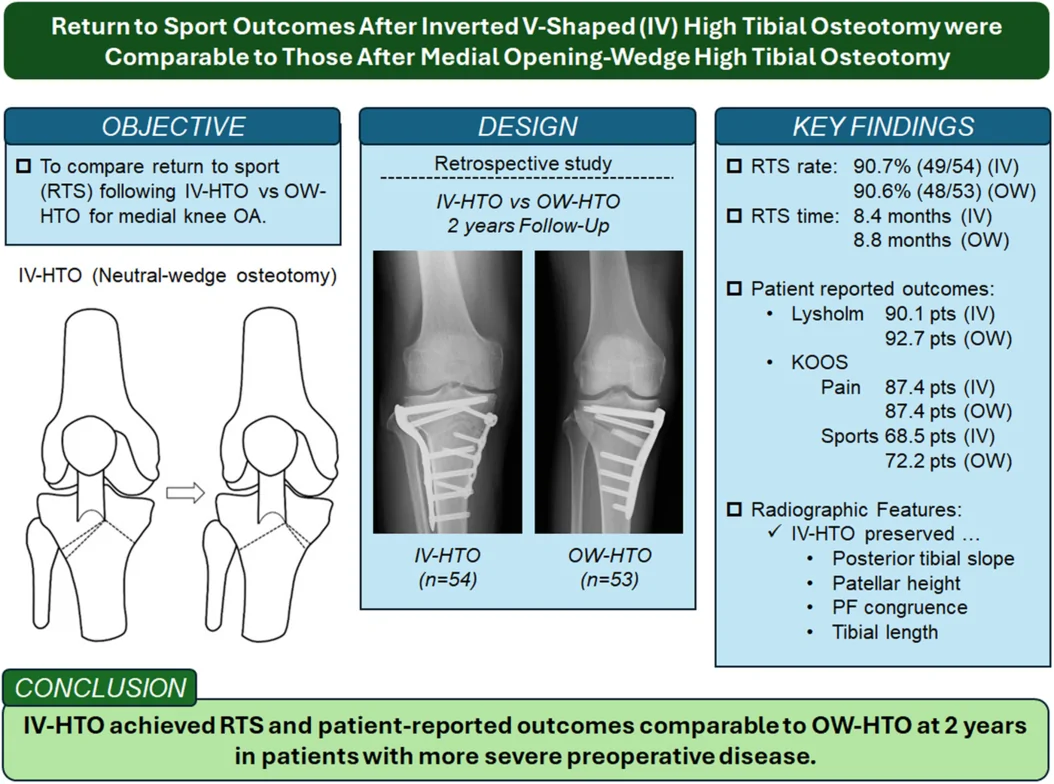

**Abbreviations**
%MA: percentage of mechanical axis
ACI: autologous chondrocyte implantation
ADL: activities of daily living
CD ratio: Caton–Deschamps ratio
CORA: centre of rotation of angulation
CW‐HTO: closing‐wedge HTO
FTA: femorotibial angle
HKA angle: hip–knee–ankle angle
HTO: high tibial osteotomy
IS ratio: Insall–Salvati ratio
IV‐HTO: inverted V‐shaped HTO
JLCA: joint line convergence angle
JOA: Japanese Orthopaedic Association
KL: Kellgren–Lawrence
KOOS: Knee Injury and Osteoarthritis Outcome Score
MCID: minimal clinically important difference
MF: microfracture
MM: medial meniscus
MPTA: medial proximal tibial angle
NW‐HTO: neutral wedge HTO
OA: osteoarthritis
OATS: osteochondral autograft
OW‐HTO: opening‐wedge HTO
PF: patellofemoral
PTS: posterior tibial slope
QoL: quality of life
RTS: return to sport
sMCL: superficial medial collateral ligament
SONK: spontaneous osteonecrosis of the knee
TAS: Tegner activity scale

## INTRODUCTION

As the number of older adults with knee osteoarthritis (OA) who desire to maintain an active lifestyle increases, understanding return to sport (RTS) outcomes following surgical intervention has become clinically important [44, 46]. High tibial osteotomy (HTO) is an established joint‐preserving procedure that unloads the medial compartment by realigning the mechanical axis [1, 9, 26]. Previous studies have reported favourable clinical outcomes and high RTS rates following medial opening‐wedge high tibial osteotomy (OW‐HTO) in patients with medial knee OA [4, 5, 7, 12, 13, 19, 21, 23, 30, 34, 37]. However, OW‐HTO is generally indicated for mild varus deformity and early‐to‐moderate‐stage medial knee OA. Therefore, evidence regarding RTS outcomes remains limited in patients with severe varus deformity and/or moderate and advanced OA, including those with concomitant patellofemoral (PF) OA.

To address these advanced cases, an inverted V‐shaped high tibial osteotomy (IV‐HTO) was developed, which is classified as a neutral‐wedge (hemi‐closing and hemi‐opening wedge) high tibial osteotomy (NW‐HTO) technique [2, 16, 49]. This procedure is designed to minimize alterations in patellar height, tibial length, posterior tibial slope (PTS) and bone mass of the tibial plateau, as the centre of alignment correction (hinge point) is located approximately at the centre of rotation of angulation (CORA) of the lower‐limb deformity [15, 16]. While previous studies have reported favourable clinical and radiographic outcomes after IV‐HTO [2, 15], RTS outcomes after IV‐HTO and direct comparisons with OW‐HTO remain limited.

The primary objective of this study was to evaluate RTS outcomes after IV‐HTO for medial knee OA and to compare them with those after OW‐HTO. The secondary objective was to compare patient‐reported and clinical outcomes, as well as radiographic parameters, between the two procedures. It was hypothesized that (1) RTS rates after IV‐HTO would be comparable to those after OW‐HTO despite more severe deformity and/or OA in the IV‐HTO cohort and (2) IV‐HTO would demonstrate smaller postoperative changes in PTS, patellar height and leg length than OW‐HTO.

## METHODS

### Study design

This retrospective study was approved by the institutional review board (No. 018‐0213). The requirement for written informed consent was waived because only existing clinical data were used, and an opt‐out approach was implemented via the hospital website. Consecutive patients who underwent HTO for medial knee OA or a varus knee with spontaneous osteonecrosis of the knee (SONK) of the medial compartment between January 2017 and December 2022 were retrospectively reviewed. Patients were allocated to IV‐HTO (IV group) or OW‐HTO (OW group) according to predefined indications based on the planned correction angle and the severity of PF‐OA, as determined preoperatively by two senior surgeons (E.K. and K.Y.) [15, 48]. IV‐HTO was indicated when a valgus correction >10° was required to shift the mechanical axis to 65% of the tibial plateau width or when PF‐OA was Kellgren–Lawrence (KL) grade ≥3 [14]. OW‐HTO was indicated when a valgus correction ≤10° was sufficient to shift the mechanical axis to 65% of the tibial plateau width and PF‐OA was KL grade ≤2.

For the RTS analysis, patients were included if they had preoperative sports participation (Tegner activity scale [TAS] ≥3 [42]) and a minimum follow‐up of 2 years. There were no restrictions on age or body mass index. Eligible knees were required to have flexion ≥130°, flexion contracture <15° and lateral‐compartment KL grade ≤1. Exclusion criteria were AP instability (i.e., clinically relevant cruciate ligament insufficiency) or coronal plane instability, defined as varus/valgus laxity >10°, as assessed by clinical examination and/or stress radiographs, follow‐up <2 years, and no expectation of returning to the same level of sports based on preoperative counselling or infection. Concomitant medial compartment procedures (e.g., cartilage or meniscal surgery) were allowed and performed as clinically indicated. Clinical and radiographic evaluations were performed preoperatively and at 2 years after surgery. RTS outcomes were analysed per patient, whereas patient‐reported and radiological outcomes were analysed per knee (index knee). For patients with bilateral procedures, only the first‐operative knee was included.

### Surgical planning and procedures

Preoperative planning was performed using the Miniaci method with a picture archiving and communication system, as reported in previous studies [16, 47]. The target postoperative alignment was set to pass through a point at 65% of the tibial plateau width from the medial edge [31, 50] (Supporting Information S1: Figure S1).

Diagnostic arthroscopy was performed initially to confirm isolated medial compartment pathology.

**Figure 1:**
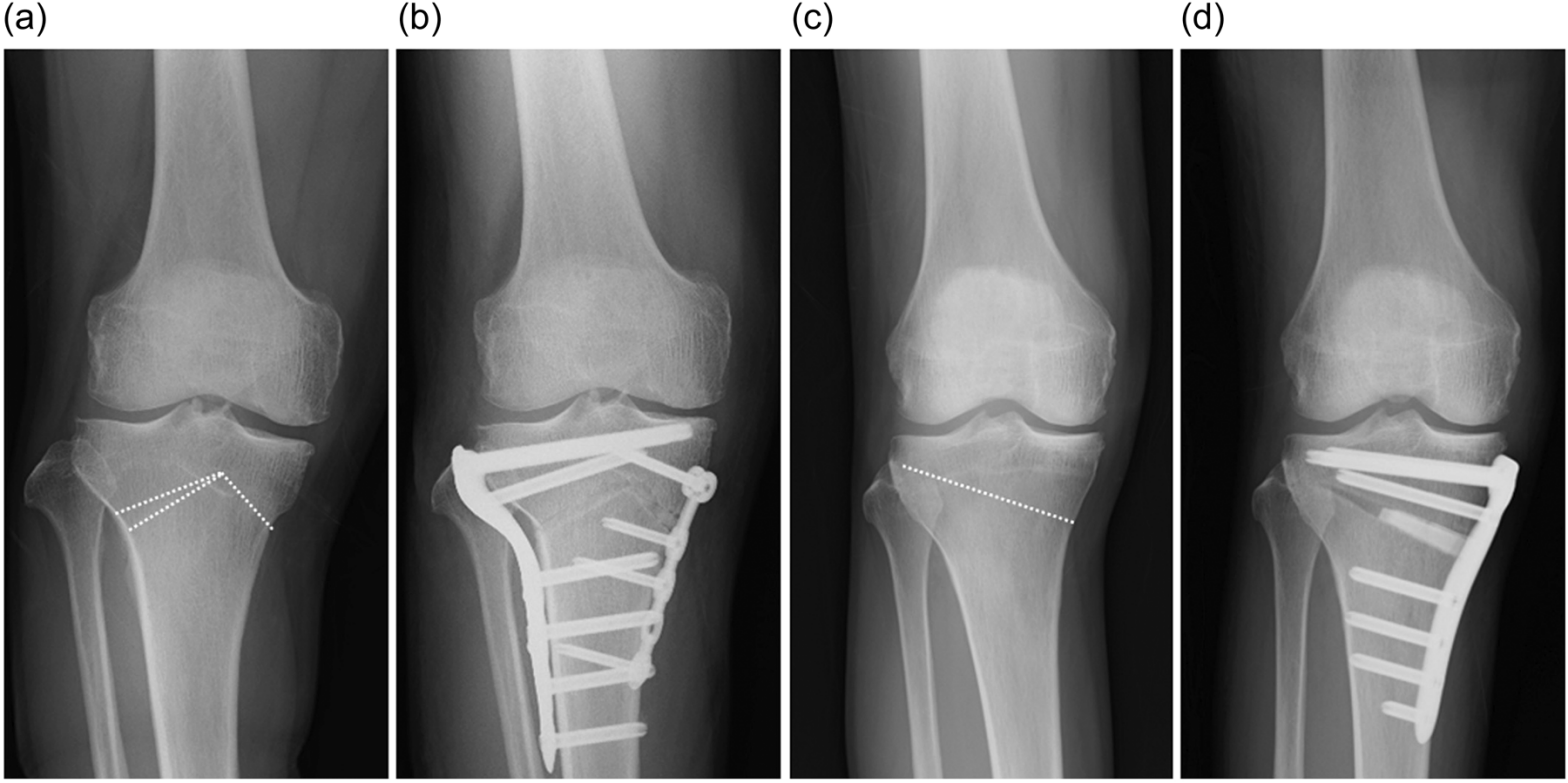
Pre‐ and post‐operative radiographs in HTO. (a) Anteroposterior radiograph before IV‐HTO. (b) Anteroposterior radiograph after IV‐HTO. (c) Anteroposterior radiograph before OW‐HTO. (d) Anteroposterior radiograph after OW‐HTO. Dotted line: osteotomy line. HTO, high tibial osteotomy; IV‐HTO, inverted V‐shaped high tibial osteotomy; OW‐HTO, opening‐wedge high tibial osteotomy.

IV‐HTO followed previously described techniques [11, 49]. Initially, an acute oblique fibular osteotomy and suture ligation procedure was performed [49]. The apex of the inverted V osteotomy was set near the attachment site of the medial edge of the patellar tendon on the tibial tubercle. A coronal ascending osteotomy and a lateral hemi‐wedge bone resection were performed, and the medial side of the tibia was then osteotomized along predrilled holes. After valgus correction, a lateral locking plate (TriS Inverted‐V Lateral HTO Plate; OSferionBiomaterials) was fixed. After implanting the resected bone block, an additional small locking plate was applied on the medial side of the tibia (Figure 1a,b).

OW‐HTO was performed using a standard medial approach and biplanar osteotomy technique as previously reported [33, 47]. After the complete release of the distal attachment of the superficial medial collateral ligament (sMCL), an ascending biplanar osteotomy of the tibial tubercle was performed. The oblique osteotomy site was gradually opened using a designed spreader based on preoperative planning, filled with two wedge‐shaped β‐tricalcium phosphate spacers (Osferion 60; OSferionBiomaterials) and fixed with a locking plate system (TriS Medial HTO plate system; OSferionBiomaterials) (Figure 1c,d).

All patients followed the same rehabilitation protocol as previously reported [15, 33]. Straight leg raising and quadriceps setting exercises, as well as active and passive knee motion exercises, were encouraged from the day after surgery. Partial weight‐bearing was permitted with crutches at 2 weeks after surgery, progressing to full weight‐bearing at 4 weeks. RTS was permitted in a stepwise manner after radiographic confirmation of osteotomy union on follow‐up radiographs obtained at ≥3 months postoperatively. Osteotomy union was defined according to previously reported criteria [48]. Progression of sports activity (including full RTS) was individualized and determined by the attending surgeon at outpatient follow‐up based on clinical symptoms (e.g., pain and swelling) and functional tolerance, rather than predefined functional testing or muscle strength measurements.

### Clinical evaluation and RTS activity

Patients were evaluated using the Japanese Orthopaedic Association (JOA) Score [32], Lysholm knee score [22] and Knee Injury and Osteoarthritis Outcome Score (KOOS) [28] preoperatively and at 2 years after surgery. Minimal clinically important difference (MCID) achievement was analysed for the Lysholm and KOOS subscales [10, 35]. The MCID thresholds were 8.9 points for the Lysholm score and 15.1, 15.4, 17.0, 11.2 and 16.5 points for KOOS symptoms, pain, activities of daily living (ADL), sports and quality of life (QoL), respectively.

RTS outcomes included sports type, time to RTS and RTS rate. TAS was used to assess the activity levels at pre‐symptomatic, preoperative and 2 years after surgery [42]. Before HTO surgery, almost all patients were forced to reduce their sports activity level due to pain. Therefore, the pre‐symptomatic level was used as the level before the pain started. In addition, sports activity was classified into four categories as described in a previous report (Supporting Information S1: Table S1) [43].

### Radiological assessment

Bilateral standing full‐length anteroposterior (AP), lateral and axial radiographs were evaluated preoperatively and at 2 years postoperatively. The degree of OA at the FT and PF joints was evaluated using the KL classification [14]. The femorotibial angle (FTA), hip–knee–ankle (HKA) angle, percentage of mechanical axis (%MA), medial proximal tibial angle (MPTA), joint line convergence angle (JLCA), whole leg length and tibial length were measured from an AP radiograph of the whole lower limb taken with a long cassette in the one‐leg standing position [24]. The PTS [45], the Insall–Salvati (IS) ratio [8] and the Caton–Deschamps (CD) ratio [3] were measured on lateral radiographs. The tilting angle was estimated in the skyline view with the knee flexed to 30° [25] (Supporting Information S1: Figure S2). One observer (Observer A) performed all radiographic measurements. For reliability, a second blinded observer (Observer B) independently measured 40 randomly selected knees for inter‐rater reliability. Observer A re‐measured the same 40 knees after an interval of at least 2 weeks to determine intra‐rater reliability. Intra‐rater and inter‐rater reliability were evaluated using ICCs.

### Statistical analysis

Statistical analyses were performed using JMP Pro 18.0 (SAS Institute Inc.). Continuous variables are presented as means and standard deviations, as appropriate, and categorical variables as numbers and percentages. Between‐group comparisons were performed using unpaired Student's *t* tests or Mann–Whitney *U* tests, according to the data distribution, and within‐group preoperative versus postoperative comparisons were performed using paired *t* tests. Categorical variables were compared using *χ* ^2^ tests or Fisher's exact tests. Because this was a retrospective study with a fixed sample size, no a priori sample size calculation was performed. Instead, the precision of the estimated RTS rate was assessed by calculating 95% confidence intervals (CIs) for a single proportion using the Clopper–Pearson exact method. With 54 patients in the IV group and 53 patients in the OW group, the study had 80% power (two‐sided *α* = 0.05) to detect a standardized between‐group difference of approximately 0.54 (Cohen's *d*). For a binary outcome with an RTS rate around 0.90, these sample sizes provide 80% power to detect an absolute between‐group difference of approximately 0.22. *p* < 0.05 was considered statistically significant. Intra‐ and inter‐rater reliabilities for radiographic measurements were evaluated using ICCs. ICCs were calculated using a two‐way model with absolute agreement. ICC values were interpreted as poor (<0.50), moderate (0.50–0.75), good (0.75–0.90) and excellent (>0.90) reliability [17].

## RESULTS

### Patient demographic data

**Figure 2:**
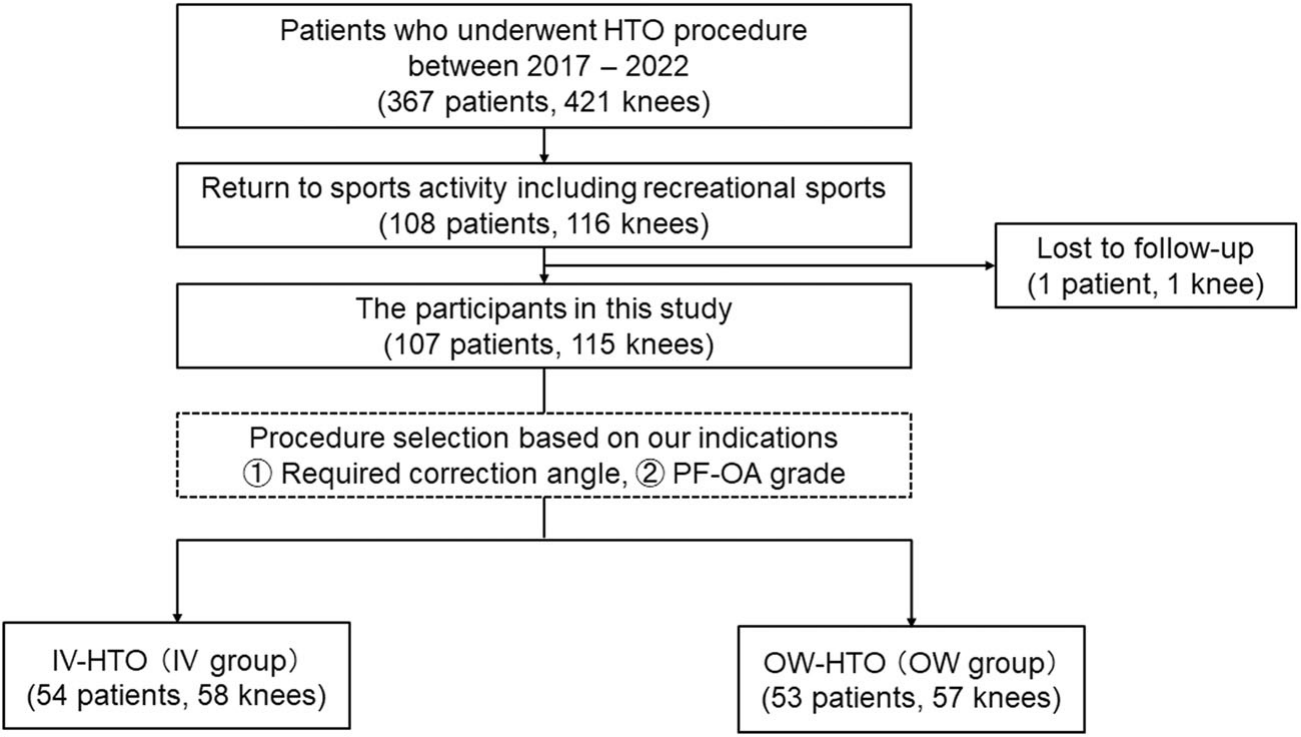
Flow diagram of patient recruitment and inclusion. HTO, high tibial osteotomy; IV‐HTO, inverted V‐shaped high tibial osteotomy; OW, opening‐wedge; PF‐OA, patellofemoral‐osteoarthritis; SONK, spontaneous osteonecrosis of the knee.

Patient selection is summarized in Figure 2. During the study period, 367 patients (421 knees) underwent HTO (IV‐HTO: 206 knees; OW‐HTO: 215 knees). Following the application of eligibility criteria, 107 patients (115 knees) were included in the final analysis (IV group: 54 patients/58 knees; OW group: 53 patients/57 knees) (Figure 2).

The total cohort had a mean age of 58.4 ± 10.3 years, mean body mass index (BMI) of 25.6 ± 4.4 kg/m^2^ and a mean follow‐up duration of 29.5 ± 7.5 months. Of these, 57 (53.3%) patients were female. Baseline characteristics are summarized in Table 1. There were no significant differences between the groups in their characteristics (all *p* > 0.05), except for preoperative OA severity. The IV group exhibited more severe FT and PF OA grades than the OW group (both *p* < 0.001).

**Table 1:** Patient demographic data.

	IV group (*n* = 54, 58 knees)	OW group (*n* = 53, 57 knees)	*p* Value
Age (years)	58.2 (10.1)	59.7 (10.5)	0.440
Male/Female (patients)	31/23	26/27	0.516
Operative side (right/left)	27/31	22/35	0.388
Unilateral/Bilateral	50/4	49/4	0.978
Height (cm)	162.6 (9.9)	163.5 (10.6)	0.640
Body weight (kg)	69.2 (15.8)	66.7 (14.9)	0.386
Body mass index (kg/m^2^)	26.1 (4.5)	24.8 (4.1)	0.095
Follow‐up duration (months)	29.6 (6.8)	29.4 (8.2)	0.897
Diagnosis (knees)	OA: 52 SONK: 6	OA: 51 SONK: 6	0.975
Femorotibial grade (no. of knees)^a^ preoperative/postoperative			
Grade 0	0/0	0/0	<0.001^b^
Grade 1	0/0	6/6	<0.001^b^
Grade 2	8/9	22/19	
Grade 3	25/25	26/28	
Grade 4	25/24	3/4	
Patellofemoral grade (no. of knees)^a^ preoperative/postoperative			
Grade 0	4/4	25/18	<0.001^b^
Grade 1	11/10	27/27	<0.001^c^
Grade 2	27/30	5/11	
Grade 3	13/12	0/1	
Grade 4	3/2	0/0	
Meniscus surgery	27	36	0.468
(No. of knees)			
MM partial resection	18	23	
MM repair	9	13	
Cartilage surgery	23	23	0.384
(No. of knees)			
MF	11	11	
OATS	11	10	
ACI	1	2	

*Note*: Data were reported as mean (SD) unless otherwise indicated.

Abbreviations: ACI, autologous chondrocyte implantation; IV, inverted V‐shaped; MF, microfracture; MM, medial meniscus; OATS, osteochondral autograft; No., number; OA, osteoarthritis; OW, opening‐wedge; SD, standard deviation; SONK, spontaneous osteonecrosis of the knee.

^a^ Determined via the Kellgren–Lawrence grading system.

^b^ Preoperative IV group versus OW group.

^c^ Postoperative IV group versus OW group.

### RTS outcomes

The distribution of sport type and impact levels before and after HTO is presented in Supporting Information S1: Tables S2 and S3.

At 2 years postoperatively, 90.7% of the cases in the IV group and 90.6% in the OW group returned to sport. The 95% CI for the RTS rate was 80.1%–96.0% in the IV group and 79.7%–95.9% in the OW group (Clopper–Pearson exact). There were no significant differences in the RTS rates between the two groups (*p* = 0.975). The average time to RTS was 8.4 months in the IV group and 8.8 months in the OW group (*p* = 0.523). Postoperative TAS were significantly higher than the preoperative scores in both groups (*p* < 0.001), with no significant differences between the pre‐symptomatic and postoperative scores in either group (all *p* > 0.05). Notably, 17 patients in the IV group (34.7%) and 17 patients in the OW group (35.4%) did not recover to their pre‐symptomatic TAS postoperatively at 2 years. These patients had higher preoperative TAS values compared to those who achieved a TAS equal to or better than their pre‐symptomatic level (IV group: *p* = 0.006, OW group: *p* = 0.024, respectively) (Table 2).

**Table 2:** RTS activity after HTO surgery.

	IV group (*n* = 54)	OW group (*n* = 53)	*p* value
RTS rate (%)	90.7 (49/54)	90.6 (48/53)	0.975
Time of RTS (months)	8.4 (3.0)	8.8 (3.4)	0.523
Tegner activity score (points)			
Pre‐symptomatic	4.8 (1.1)	5.0 (1.2)	0.358
Preoperative	2.8 (1.2)	3.2 (0.9)	0.034
Postoperative	4.3 (1.1)^a^	4.6 (1.2)^a^	0.193
Sports activity level pre/post (no.)			
Low	16/21	15/17	0.982^b^
Potentially low	9/9	8/8	0.481^c^
Intermediate	5/2	6/6	
High	24/17	24/17	

*Note*: Data were reported as mean (SD) unless otherwise indicated.

Abbreviations: HTO, high tibial osteotomy; IV, inverted V‐shaped; OW, opening‐wedge; RTS, return to sport; SD, standard deviation.

^a^ A significant difference between pre‐symptomatic, preoperative and postoperative Tegner activity score.

^b^ Preoperative IV group versus OW group.

^c^ Postoperative IV group versus OW group.

Of the 10 patients (five in each group) who could not RTS after surgery, nine patients (five in the IV group and four in the OW group) had participated in intermediate‐ and high‐activity sports (e.g., baseball, skiing, judo, bicycle racing) before surgery.

### Clinical evaluation

The functional knee score (JOA score), Lysholm score and KOOS significantly improved after both types of HTO surgery compared to preoperative values (*p* < 0.001) (Table 3). Clinical outcomes were comparable between groups, except for the preoperative JOA score, which was significantly lower in the IV group (*p* = 0.021) (Table 3).

**Table 3:** Clinical outcomes before and after HTO surgery.

	IV group			OW group			Between‐group *p* value	
	Preop	Postop	*p* value	Preop	Postop	*p* value	Preop	Postop
JOA score (points)	66.4 (14.2)	90.3 (12.9)	<0.001	72.5 (14.0)	92.0 (9.2)	<0.001	0.021	0.406
Lysholm score (points)	62.0 (15.9)	90.1 (12.7)	<0.001	64.6 (15.9)	92.7 (8.8)	<0.001	0.375	0.210
KOOS (points)								
Symptom	65.3 (16.5)	85.0 (11.9)	<0.001	62.5 (22.1)	86.9 (13.9)	<0.001	0.488	0.458
Pain	61.5 (16.1)	87.4 (10.5)	<0.001	61.5 (20.2)	87.4 (13.6)	<0.001	0.996	0.999
ADL	73.4 (16.4)	91.0 (8.6)	<0.001	69.4 (20.1)	92.9 (7.7)	<0.001	0.282	0.225
Sports	39.1 (23.5)	68.5 (23.1)	<0.001	33.8 (24.4)	72.2 (25.6)	<0.001	0.283	0.431
QoL	37.9 (20.5)	68.3 (21.5)	<0.001	30.1 (19.1)	73.9 (21.5)	<0.001	0.060	0.190

*Note*: Data were reported as mean (SD) unless otherwise indicated. Between‐group *p* values compared IV versus OW at each time point.

Abbreviations: ADL, activities of daily living; IV HTO, inverted V‐shaped; JOA, Japanese Orthopaedic Association; KOOS, Knee Injury and Osteoarthritis Outcome Score; OW, opening‐wedge; postop, postoperative; preop, preoperative; QoL, quality of life.

MCID responder rates for the Lysholm score and each KOOS subscale did not differ significantly between the groups (Table 4).

**Table 4:** Proportion of patients achieving the MCID in patient‐reported outcome measures.

Outcome	MCID threshold (points)	IV group	OW group	*p* value
Lysholm score (points)	8.9	48/54 (88.9%)	51/53 (96.2%)	0.149
KOOS (points)				
Symptom	15.1	31/54 (57.4%)	32/53 (60.4%)	0.755
Pain	15.4	41/54 (75.9%)	36/53 (67.9%)	0.357
ADL	17.0	30/54 (55.6%)	37/53 (69.8%)	0.128
Sports	11.2	39/54 (72.2%)	39/53 (73.6%)	0.874
QoL	16.5	39/54 (72.2%)	44/53 (83.0%)	0.181

*Note*: Data were reported as number of patients achieving MCID/total number of patients (%). Percentages are calculated using the number of patients with complete pre‐ and postoperative data for each score.

Abbreviations: ADL, activities of daily living; IV, inverted V‐shaped; KOOS, Knee Injury and Osteoarthritis Outcome Score; MCID, minimal clinically important difference; OW, opening‐wedge; QoL, quality of life.

### Complications

Intraoperatively, lateral hinge fractures occurred in two patients in the OW group (2/53, 3.8%); no intraoperative complications were observed in the IV group. Postoperatively, delayed union (>4 months) occurred in three patients in the IV group (3/54, 5.6%) and in one patient in the OW group (1/53, 1.9%); bone healing was achieved in all cases through a reduction in activity level. In the OW group, one superficial infection (1/53, 1.9%) occurred and was treated with antibiotic therapy and early surgical irrigation and debridement. One case of delayed wound healing (1/53, 1.9%) was also observed in the OW group and was managed with continued local wound care without further surgical intervention.

### Radiological assessment

Intra‐ and inter‐rater reliability for all radiographic parameters was excellent　(Supporting Information S1: Table S4). The radiographic measurements are summarized in Table 5. Preoperative coronal lower leg alignments (the FTA, HKA angle, %MA and MPTA) were significantly higher in the IV group than in the OW group (*p* < 0.001). Postoperatively, these parameters significantly changed when compared with the preoperative values in both groups (*p* < 0.001), and no significant differences were observed between the groups. Concerning the leg length, the postoperative whole leg length was significantly increased in the OW group (*p* < 0.001), but showed no significant change in the IV group. In the lateral radiographic view, the postoperative PTS was significantly increased compared with the preoperative value in the OW group (*p* < 0.001), whereas no significant change was observed in the IV group (*p* = 0.452). Regarding patella height, the IS and CD ratio significantly decreased in the OW group postoperatively (*p* < 0.001), while no significant changes were observed between the pre‐ and postoperative values in the IV group (all *p* > 0.05). On the axial view of the PF joint, the IV group showed a significant decrease in the tilting angle postoperatively (*p* < 0.001), whereas no significant change was seen in the OW group (*p* = 0.052).

**Table 5:** Radiographic evaluation before and after HTO surgery.

	IV group			OW group			Between‐group *p* value	
	Preop	Postop	*p* value	Preop	Postop	*p* value	Preop	Postop
FTA (°)	181.7 (3.3)	170.0 (3.0)	<0.001	178.9 (2.4)	169.3 (1.5)	<0.001	<0.001	0.105
HKA angle (°)	−7.7 (3.4)	3.9 (2.8)	<0.001	−4.8 (2.7)	4.5 (1.5)	<0.001	<0.001	0.114
Mechanical axis (%)	14.1 (15.6)	63.8 (10.8)	<0.001	27.9 (10.0)	65.5 (5.6)	<0.001	<0.001	0.292
MPTA (°)	82.4 (3.0)	93.1 (1.9)	<0.001	84.4 (2.0)	92.7 (1.4)	<0.001	<0.001	0.220
JLCA (°)	3.3 (1.8)	2.0 (1.4)	<0.001	2.1 (1.2)	1.3 (1.0)	<0.001	<0.001	0.002
PTS (°)	9.1 (2.7)	9.0 (2.7)	0.452	9.3 (2.3)	10.2 (2.4)	<0.001	0.708	0.011
Whole leg length (mm)	791.5 (56.0)	792.3 (55.7)	0.241	800.3 (59.2)	807.9 (59.4)	<0.001	0.416	0.151
Tibial length (mm)	351.6 (28.1)	351.6 (27.6)	0.986	352.6 (29.1)	361.3 (30.7)	<0.001	0.857	0.079
IS ratio	1.04 (0.12)	1.02 (0.11)	0.063	1.07 (0.12)	0.99 (0.12)	<0.001	0.163	0.096
CD ratio	0.99 (0.12)	0.98 (0.13)	0.142	1.03 (0.13)	0.88 (0.14)	<0.001	0.100	<0.001
Tilting angle (°)	7.1 (4.2)	4.9 (2.8)	<0.001	4.9 (2.9)	5.2 (3.1)	0.052	0.002	0.577

*Note*: Data were reported as mean (SD) unless otherwise indicated. Between‐group *p* values compared IV versus OW at each time point.

Abbreviations: CD ratio, Caton–Deschamps ratio; FTA, femorotibial angle; HKA angle, hip–knee–ankle angle; HTO, high tibial osteotomy; IS ratio, Insall–Salvati ratio; JLCA, joint line convergence angle; MPTA, medial proximal tibial angle; postop, postoperative; preop, preoperative; PTS, posterior tibial slope; SD, standard deviation.

## DISCUSSION

The present study demonstrated that 90.7% of patients in the IV group returned to sport at an average of 8.4 months after surgery. Similarly, 90.6% of patients returned to sport at a mean of 8.8 months after OW‐HTO, indicating comparable RTS outcomes between the two techniques. Notably, although the preoperative OA grade and varus deformity were more severe in the IV group than in the OW group, postoperative clinical outcomes, including RTS, functional scores and activity levels, were similar between the groups. Radiographically, postoperative PTS, patellar height and leg length significantly changed in the OW group, whereas no significant changes were observed in the IV group. To date, this is the first study to report RTS outcomes after IV‐HTO in patients with severe medial knee OA.

The RTS rate observed in this study is consistent with previous reports after HTO. Previous studies on OW‐HTO have shown RTS rates ranging from 80% to 90% across various age groups and activity levels [4, 5, 12, 21, 34, 36]. In contrast, there are limited reports on RTS after closing‐wedge HTO (CW‐HTO) and NW‐HTO. Nakashima et al. [29] reported that 80% of patients who underwent hybrid CW‐HTO returned to sport at a mean of 7.2 months. These findings suggest that IV‐HTO achieves high RTS rates comparable to conventional OW‐HTO, even in patients with advanced OA and severe varus deformity.

Several radiographic features of IV‐HTO may be biomechanically favourable for RTS. First, IV‐HTO preserved patellar height and improved PF joint congruence [15]. Alterations in patellar height, particularly patella baja, are associated with anterior knee pain and increased PF joint loading [40, 41]. Although PF‐specific symptoms were not directly evaluated, preservation of patellar height may be advantageous for high‐demand activities. Second, IV‐HTO minimized changes in PTS, whereas PTS increased after OW‐HTO in this study. Biomechanical studies suggest that increased PTS may lead to greater anterior tibial translation, increased anterior cruciate ligament strain and altered meniscal loading [6, 20, 39], which could potentially hinder RTS. Third, minimal change in leg length is a potential benefit of IV‐HTO [2, 15]. Leg length discrepancy in athletes is associated with an increased risk of sports injuries, including stress fractures and low back pain [18, 38]. While the direct impact of these radiographic advantages on RTS requires further confirmation, IV‐HTO appears to maintain a more physiological joint environment of the knee postoperatively.

Nevertheless, 34.7% of the IV group did not achieve a postoperative TAS equal to or greater than the pre‐symptomatic score. The results were similar to a previous systematic review [4], which reported that 21.4% of patients following HTO could not RTS at an equal or greater level postoperatively. Several authors have highlighted that higher preoperative activity levels and participation in high‐impact sports are associated with difficulty returning to the original level of sport [5, 13, 27, 37]. In this study, patients who failed to recover their pre‐symptomatic TAS often participated in high‐impact activities preoperatively, suggesting that a full return to high‐intensity sports remains challenging regardless of the surgical technique.

The present study demonstrated that, although preoperative varus alignment and FT and PF OA grades were more severe in the IV group than in the OW group, clinical outcomes and RTS rates in the IV group were comparable to those in the OW group. The IV‐HTO procedure allows large correction with minimal bone resection, restores PF congruence and limits adverse effects on PTS and leg length [2, 15]. These findings suggest that IV‐HTO may be a surgical option for active patients with advanced OA who wish to RTS.

Several limitations should be acknowledged. First, this was a retrospective study, and the choice between IV‐HTO and OW‐HTO was based on preoperative deformity and PF‐OA severity, introducing selection bias. Second, although baseline characteristics were compared between groups, residual confounding cannot be entirely excluded and multivariable adjustment or matching was not performed. Third, detailed information on the frequency, intensity or duration of sports participation after RTS was not collected, and anterior knee pain, PF‐specific symptoms or knee instability were not evaluated, despite the discussion of PF alignment and PTS. Finally, this study reports short‐term outcomes; therefore, whether activity levels and sports participation can be maintained at longer‐term follow‐up remains unclear. Larger prospective comparative studies with longer follow‐up are needed to further clarify the role of IV‐HTO in active patients with severe medial knee OA.

## CONCLUSION

IV‐HTO achieved RTS and patient‐reported outcomes comparable to OW‐HTO at 2 years in patients with more severe preoperative disease.

## AUTHOR CONTRIBUTIONS

Taku Ebata and Eiji Kondo conceived the project and designed the experiments. Taku Ebata, Koji Yabuuchi, Koji Iwasaki, Dai Sato and Masatake Matsuoka collected and assembled data. Taku Ebata, Tomohiro Onodera and Eiji Kondo analysed and interpreted the data. Eiji Kondo, Kazunori Yasuda and Tomonori Yagi recruited patients. Taku Ebata and Eiji Kondo prepared the draft of the manuscript. Eiji Kondo, Kazunori Yasuda and Norimasa Iwasaki supervised the project. All authors critically reviewed the manuscript and provided their comments.

## CONFLICT OF INTEREST STATEMENT

The authors declare no conflicts of interest.

## ETHICS STATEMENT

This study protocol was approved by the institutional review board of Hokkaido University Hospital (approval ID: 018‐0213). The requirement for written informed consent was waived because only existing clinical data were used, and an opt‐out approach was implemented via the hospital website.

## Supporting information

Final Supplementary Materials.

## Data Availability

The data that support the findings of this study are available on request from the corresponding author. The data are not publicly available due to privacy or ethical restrictions.
