# Supplementary material for: Return to sport outcomes after inverted V‐shaped (IV) high tibial osteotomy were comparable to those after medial opening‐wedge high tibial osteotomy, even though the IV cohort had more severe preoperative disease
Source: J Exp Orthop. 2026 Feb 23;13(1):e70667. doi: 10.1002/jeo2.70667 (PMC12928534; doi:10.1002/jeo2.70667)
Supplement: Supplementary file 1 — Final Supplementary Materials. [file JEO2-13-e70667-s001.docx]

**Supplementary Figure 1. Preoperative planning**


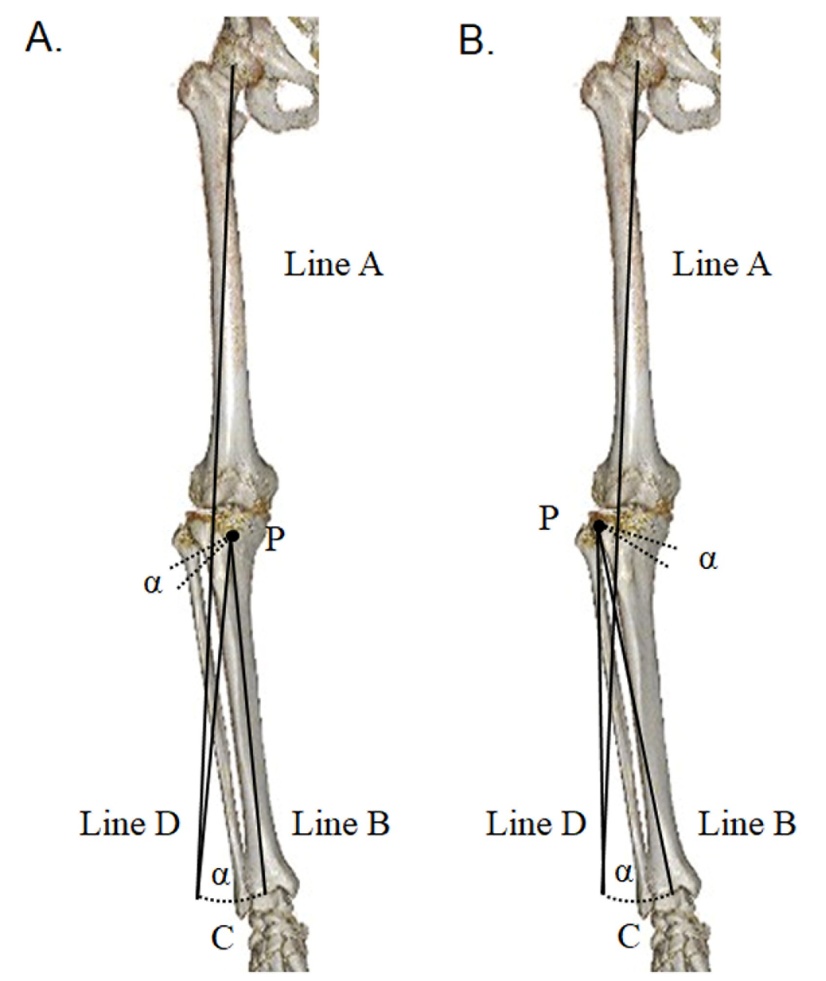


1. In the IV group, the hinge point ‘P’ is located approximately at the medial edge of the tibial tubercle.
2. In the OW group, the hinge point ‘P’ is located approximately 5 mm medial from the proximal tibiofibular joint.

First, a ‘line A’ is drawn from the centre of the femoral head through the point 65% lateral from the medial edge of the tibial plateau on the lateral tibial plateau. Second, a ‘line B’ is drawn from hinge point P to the centre of the talar dome. Then an arc C, the centre and the radius of which are the hinge point P and line B, respectively, is drawn so that the arc is across line A. Next, a ‘line D’ is drawn from hinge point P to the crossing point between line A and arc C. The angle (alpha) formed between line B and line D provides the correction angle.

Abbreviation: IV, Inverted V-shaped HTO; OW, Opening-wedge HTO.

**Supplementary Figure 2. Radiographic assessments**


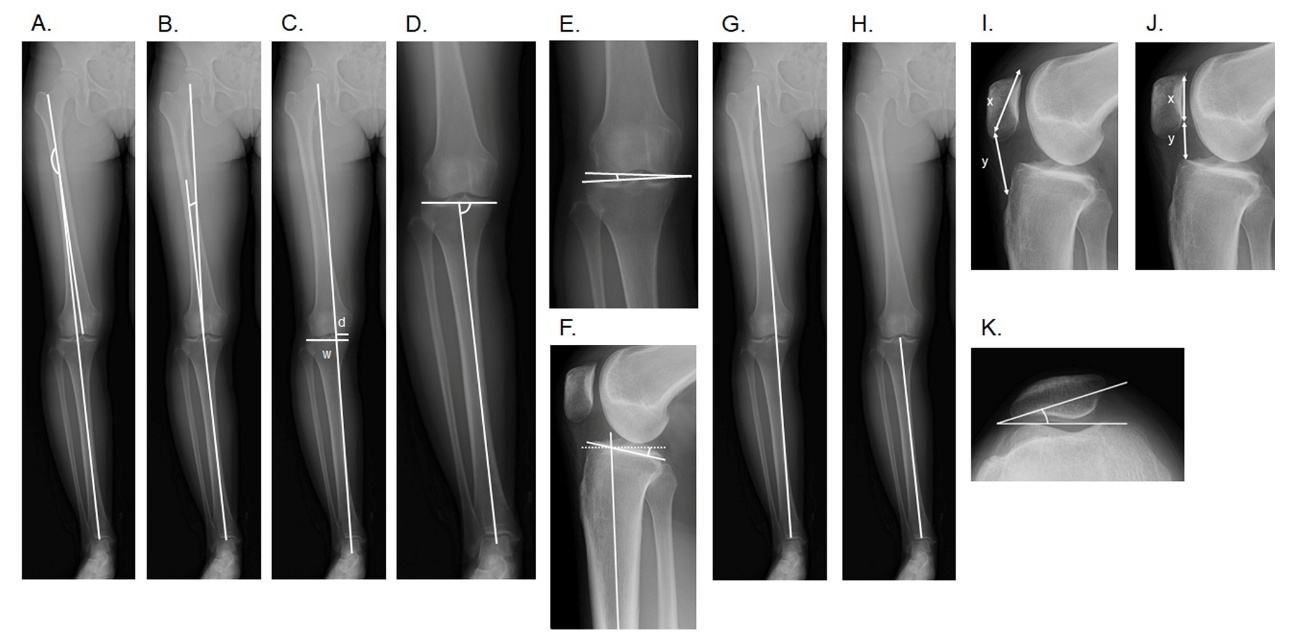


A) Femorotibial angle (FTA).　The FTA was defined as the angle between the anatomical axis of the femoral shaft and　the axis of the tibial shaft on the fibular side.

B) Hip–knee–ankle angle (HKA). The HKA was defined as the angle between the　femoral mechanical axis and the tibial mechanical axis.

C) Mechanical axis of the lower limb. A mechanical axis line was drawn from the centre of the femoral head to the middle point of the proximal talar joint surface. The mechanical axis percentage was defined as the horizontal distance from the mechanical axis line to the medial edge of the tibial plateau (d), divided by the width of the tibial plateau (w).

D) Medial proximal tibial angle (MPTA). The MPTA was defined as the angle between the proximal tibial joint line and the mechanical axis of the tibial shaft.

E) Joint line convergence angle (JLCA). The JLCA was defined as the angle between the distal femoral joint line and the proximal tibial joint line.

F) Posterior tibial slope (PTS). The PTS was defined as the angle between the line perpendicular to the mid-diaphysis of the tibia and the posterior inclination of the medial tibial plateau.

G) Entire leg length. The entire leg length was defined as the distance between the top of the femoral head and the centre of the tibial plafond.

H) The tibial length was defined as the distance between the centre of the proximal tibia and the centre of the tibial plafond.

I) Insall-Salvati (IS) ratio. The IS ratio was defined as a ratio of the length of the patella tendon (the distance between the inferior pole of the patella and the tibial tubercle (x)) divided by the patellar length (y).

J) Caton-Deschamps (CD) ratio. The CD ratio was defined as a ratio of the distance (x) between the inferior pole of the patella and the antero-proximal edge of the tibial plateau divided by the patellar length (y).

K) Tilting angle. The tilting angle was defined as the angle between the line intersecting the widest bony structure of the patella and the line tangentially passing the anterior surface of the femoral condyles.

**Supplementary Table 1.** The classification according to the level of impact on joints

| Level of impact on joints | Sports examples |
| --- | --- |
| Low | Cycling  Calisthenics  Golf  Swimming  Walking,  Ballroom dancing  Classical ballet  Water aerobics |
| Potentially Low | Bowling  Fencing  Rowing  Isokinetic weightlifting  Sailing  Fast walking  Hiking  Table tennis  Cross-country skiing  Jazz dancing and Ballet  Bicycling |
| Intermediate | Free weightlifting  Horseback riding  Ice skating  Rock climbing  Low-impact aerobics  Doubles tennis  In-line skating  Downhill skiing |
| High | Basketball/volleyball  Football  Handball/racketball  Jogging/Running  Lacrosse  Soccer  Singles tennis  Badminton  Water skiing  Karate |

**Supplementary Table 2.** Sports activity before and after IV-HTO surgery.

| Sports activity level | Preoperative  (No. of patients) | Postoperative  (No. of patients) |
| --- | --- | --- |
| Low | Swimming (5)  Golf (6)  Cycling (2)  Ballroom dancing (1)  Classical ballet (1)  Gym (1) | Swimming (6)  Golf (6)  Cycling (6)  Yoga (1)  Classical ballet (1)  Gym (1) |
| Potentially Low | Fast walking (1)  Hiking (7)  Table tennis (1) | Hiking (8)  Table tennis (1) |
| Intermediate | Doubles tennis (4)  Ski (1) | Doubles tennis (2) |
| High | Jogging/Running (18)  Baseball (2)  Badminton (2)  Singles tennis (1)  Karate (1) | Jogging/Running (15)  Badminton (1)  Karate (1) |

**Supplementary Table 3.** Sports activity before and after OW-HTO surgery.

| Sports activity level | Preoperative  (No. of patients) | Postoperative  (No. of patients) |
| --- | --- | --- |
| Low | Swimming (1)  Golf (6)  Cycling (3)  Ballroom dancing (3) Yoga (1)  Classical ballet (1)  Gym (1) | Swimming (1)  Golf (5)  Cycling (4)  Ballroom dancing (4)  Yoga (1)  Gym (2) |
| Potentially Low | Hiking (8) | Hiking (8) |
| Intermediate | Doubles tennis (1)  Ski (4) | Doubles tennis (1)  Ski (5) |
| High | Jogging/Running (12)  Volleyball (3)  Soccer (1)  Badminton (1)  Single tennis (4)  Bicycle Racing (1)  Judo (2) | Jogging/Running (10)  Volleyball (2)  Soccer (1)  Badminton (1)  Single tennis (3) |

**Supplementary Table 4. Intra- and inter-rater reliability for radiographic measurements**.

Data were reported as ICC (95% confidence interval). ICCs were calculated for single-measure absolute agreement using a two-way model. Intra-rater reliability: Observer 1 repeated measurements after an interval of ≥2 weeks. Inter-rater reliability: measurements from two observers were compared.

Abbreviations: ICCs, Intraclass correlation coefficients; FTA, Femorotibial angle; HKA angle, Hip-Knee-Ankle angle; MPTA, Medial proximal tibial angle; JLCA, Joint line convergence angle; PTS, Posterior tibial slope angle; IS ratio, Insall-Salvati ratio; CD ratio, Caton-Deschamps ratio

|  | Intra-rater ICC | Inter-rater ICC |
| --- | --- | --- |
| FTA | 0.970 (0.920-0.987) | 0.949 (0.891-0.971) |
| HKA angle | 0.957 (0.879-0.983) | 0.899 (0.780-0.939) |
| Mechanical axis | 0.947 (0.888-0.978) | 0.969 (0.935-0.984) |
| MPTA | 0.972 (0.917-0.988) | 0.953 (0.848-0.976) |
| JLCA | 0.971 (0.940-0.988) | 0.966 (0.927-0.984) |
| PTS | 0.982 (0.961-0.990) | 0.920 (0.870-0.948) |
| Whole leg length | 0.966 (0.946-0.986) | 0.957 (0.923-0.974) |
| Tibial length | 0.941 (0.908-0.964) | 0.933 (0.861-0.973) |
| IS ratio | 0.910 (0.857-0.942) | 0.952 (0.920-0.969) |
| CD ratio | 0.978 (0.958-0.990) | 0.972 (0.947-0.983) |
| Tilting angle | 0.959 (0.933-0.978) | 0.947 (0.902-0.970) |
